# Behavioral Reserve in Behavioral Variant Frontotemporal Dementia

**DOI:** 10.3389/fnagi.2022.875589

**Published:** 2022-06-20

**Authors:** Su Hong Kim, Yae Ji Kim, Byung Hwa Lee, Peter Lee, Ji Hyung Park, Sang Won Seo, Yong Jeong

**Affiliations:** ^1^Graduate School of Medical Science and Engineering, Korea Advanced Institute of Science and Technology, Daejeon, South Korea; ^2^KAIST Institute for Health Science Technology, Korea Advanced Institute of Science and Technology, Daejeon, South Korea; ^3^Program of Brain and Cognitive Engineering, Korea Advanced Institute of Science and Technology, Daejeon, South Korea; ^4^Department of Neurology, Samsung Medical Center, Sungkyunkwan University, Seoul, South Korea; ^5^Neuroscience Center, Samsung Medical Center, Seoul, South Korea; ^6^Samsung Alzheimer Research Center, Samsung Medical Center, Seoul, South Korea; ^7^Department of Health Science and Technology, Samsung Advanced Institute for Health Sciences and Technology (SAIHST), Sungkyunkwan University, Seoul, South Korea; ^8^Department of Intelligent Precision Healthcare Convergence, Samsung Advanced Institute for Health Sciences and Technology (SAIHST), Sungkyunkwan University, Seoul, South Korea; ^9^Department of Bio and Brain Engineering, Korea Advanced Institute of Science and Technology, Daejeon, South Korea

**Keywords:** behavioral variant frontotemporal dementia, behavior reserve, neural correlates, brain network, MRI

## Abstract

“Reserve” refers to the individual clinical differences in response to a neuropathological burden. We explored the behavioral reserve (BR) and associated neural substrates in 40 participants with behavioral variant frontotemporal dementia (bvFTD) who were assessed with the frontal behavioral inventory (FBI) and magnetic resonance imaging. Because neuroimaging abnormality showed a high negative correlation with the FBI negative (but not positive) symptom scores, we developed a linear model only to calculate the nBR (BR for negative symptoms) marker using neuroimaging abnormalities and the FBI score. Participants were divided into high nBR and low nBR groups based on the nBR marker. The FBI negative symptom score was lower in the high nBR group than in the low nBR group having the same neuroimaging abnormalities. However, the high nBR group noted a steeper decline in cortical atrophy and showed less atrophy in the left frontotemporal cortices than the low nBR group. In addition, the fractional anisotropy (FA) values were greater in the high nBR than in the low nBR group, except in the sensory-motor and occipital areas. We identified an nBR-related functional network composed of bilateral frontotemporal areas and the left occipital pole. We propose the concept of BR in bvFTD, and these findings can help predict the disease progression.

## Introduction

Frontotemporal dementia (FTD) is the second most common cause of early-onset dementia after Alzheimer’s Disease (AD); also, it is associated with progressive degeneration of the frontal and temporal lobes (Bang et al., 2015). Behavioral variant FTD (bvFTD) is the most common subtype of FTD and is characterized by personality change and social dysfunction (Rascovsky et al., 2011). The severity of behavioral manifestations of bvFTD is commonly evaluated using the frontal behavioral inventory (FBI), which is a caregiver-based questionnaire consisting of 12 positive and 12 negative behavioral symptoms (Kertesz et al., 1997, 2000; Milan et al., 2008).

The concept of “reserve” refers to individual differences in the capacity to withstand the pathological burden of neurodegenerative diseases (Katzman et al., 1988). Compared with people with a low reserve, people with a higher reserve can cope with a greater pathological burden before the onset of symptoms; they may show a less severe clinical presentation under similar pathological burdens. However, if the pathological burden increases, participants with a high reserve show a faster decline in the clinical presentation than those with a low reserve (Stern, 2012; Barulli and Stern, 2013). Cognitive reserve (CR) is a widely accepted concept in AD (Stern, 2012; Lee et al., 2019). Moreover, the concept of motor reserve (MR) has recently emerged in Parkinson’s Disease (PD) (Chung et al., 2020a,b). Previous research has suggested that CR is an environmental factor that contributes to heterogeneous cognitive function mediated by the neuroanatomical structure, metabolism, or cerebral blood flow; the precise mechanism remains unclear (Placek et al., 2016; Maiovis et al., 2018; Beyer et al., 2021).

Using FBI scores and statistical modeling, Borroni et al. (2012) identified four behavioral subgroups in participants with bvFTD, namely “disinhibited,” “apathic,” “language,” and “aggressive.” Furthermore, Premi et al. (2013) proposed the behavioral reserve (BR) hypothesis in FTD with cerebral single-photon emission computed tomography (SPECT); this is similar to the concepts of CR in AD and of MR in PD. These studies revealed that educational attainment was the only measure associated with a disinhibited phenotype. These studies also observed greater hypoperfusion in the right inferior frontal gyrus, left medial frontal gyrus, and right caudate in those with a higher education level than those with a lower education level.

The degree of behavioral manifestation varies in participants with bvFTD, even with similar degrees of cortical atrophy or white matter (WM) destruction. In this study, we have proposed a concept of BR to understand these individual behavioral differences. We have suggested a novel model to conceptualize BR based on the original definition of reserve, using T1 imaging and fractional anisotropy (FA) as surrogates of the neuropathological burden of gray matter (GM) and WM, respectively. Subsequently, we have tested whether BR can explain the heterogeneous clinical presentation of bvFTD.

Neural compensation, one of the neural reserve mechanisms, is a brain network newly developed to compensate for the disruption of the pre-existing brain network due to the disease pathology. In this respect, we also investigated the neural correlates and the associated networks of BR by estimating the BR of each participant with bvFTD based on their FBI score and neuroimaging abnormalities. Then, we identified the BR-associated functional brain networks using network-based statistics (NBSs) analysis.

## Materials and Methods

### Ethics Approval Statement

This study was approved by the Institutional Review Board of the Samsung Medical Center. Written informed consent was obtained from the caregivers of all participants prior to conducting the study procedures.

### Participants

**FIGURE 1:**
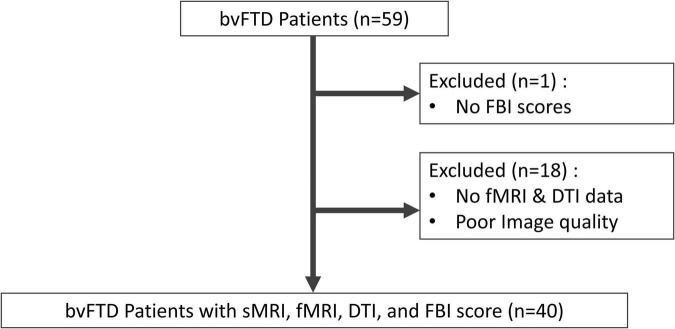
Flowchart of the study participants and their enrolment. bvFTD, behavioral variant frontotemporal dementia; FBI, frontal behavioral inventory; fMRI, functional magnetic resonance imaging; DTI, diffusion tensor imaging; sMRI, structural magnetic resonance imaging.

In this study, 59 participants presenting to the Neurology Department of the Samsung Medical Center between January 2011 and September 2018 were screened. They were diagnosed with bvFTD based on the clinical criteria proposed by the International Behavioral Variant Frontotemporal Dementia Criteria Consortium for probable bvFTD (Rascovsky et al., 2011). All participants were evaluated using the FBI and through comprehensive interviews; they also underwent magnetic resonance imaging (MRI), including high-resolution T1-weighted MRI, resting-state functional MRI (rs-fMRI), and diffusion tensor imaging (DTI). Among the 59 participants screened, 1 without FBI scores and 18 without rs-fMRI and DTI scans were excluded. Finally, 40 participants were included in this study (Figure 1).

Blood tests, including a complete blood count, blood chemistry tests, vitamin B12/folate test, thyroid function test, and serological tests for syphilis, were performed to exclude the secondary causes of dementia. In addition, conventional brain MRI confirmed the absence of severe WM diseases and structural lesions, such as traumatic brain injuries, brain tumors, and hydrocephalus.

The basic demographical data were obtained. Considering that the years of education and occupational complexity have been widely accepted as the most relevant CR estimates, we also obtained data on these parameters to test whether they are valid estimates even for BR. Occupational complexity was measured using the Dictionary of Occupational Titles (DOT), which evaluated the complexity of dealing with data (0–6 points), people (0–8 points), and things (0–7 points) (United States Department of Labor and United States Employment Service and Center, 2006). In the DOT ratings, a lower score indicates a higher occupational complexity. Thus, we investigated the correlations between the years of education, occupational complexity, and BR markers.

### Frontal Behavioral Inventory Scores

Behavioral symptoms were quantified using the FBI, which is a caregiver-based behavioral questionnaire consisting of 12 positive and 12 negative symptoms (Kertesz et al., 1997, 2000; Milan et al., 2008). The positive symptoms include obsessions, hoarding, inappropriateness, excessive jocularity, impulsivity, restlessness, irritability, aggression, hyperorality, hypersexuality, utilization behavior, and incontinence. The negative symptoms include apathy, aspontaneity, emotional flatness, inflexibility, disorganization, inattention, personal neglect, loss of insight, logopenia, aphasia, comprehensive deficit, and alien hand. Each item is scored 0–3 points (0: no symptom, 1: mild, 2: moderate, and 3: severe); thus, the maximum score is 72. The behavioral score was newly defined so that it was directly proportional to the behavioral performance, reflecting the concept of reserve well and making it easier to understand. The positive and negative behavioral score was calculated by subtracting the FBI score of the positive items or the FBI score of the negative items from the maximum score (36 points); retrospectively, a lower score indicated severe symptoms. The FBI symptoms were categorized into the following four phenotypes (Borroni et al., 2012): (1) disinhibited phenotype (which comprised loss of insight, obsession, hoarding, excessive jocularity, impulsivity, restlessness, hyperorality, and utilization behavior), (2) apathetic phenotype (which comprised apathy and aspontaneity), (3) aggressive phenotype (which comprised inflexibility, irritability, and aggression), and (4) language phenotype (which comprised logopenia, aphasia, comprehensive deficit, and alien hand). We explored the correlations between the scores of each phenotype and the neuroimaging abnormalities.

### Magnetic Resonance Imaging Acquisition

An Achieva 3.0-Tesla MRI scanner (Philips, Best, Netherlands) was used to obtain all sequences. A three-dimensional (3D), T1-weighted turbo field echo image was obtained with the following parameters: sagittal slice thickness, 1.0 mm (over contiguous slices with 50% overlap); no gap; repetition time (TR), 9.9 ms; echo time (TE), 4.6 ms; flip angle, 8°; and matrix size, 240 × 240 [reconstructed to 480 × 480 over a field of view (FOV) of 240 mm]. DTI images were obtained with sets of axial diffusion-weighted, single-shot, echo-planar images with the following parameters: acquisition matrix, 128 × 128; voxel size, 1.72 mm × 1.72 mm × 2 mm; axial slices, 70; FOV, 220 mm × 220 mm; TE, 60 ms; TR, 7,696 ms; flip angle, 90°; slice gap, 0 mm; and b-factor, 600 smm^–2^. DTI images were acquired in 45 different directions using the baseline image without weighting (0, 0, 0). An rs-fMRI sequence was obtained using a gradient echo-planar imaging pulse sequence with the following parameters: acquisition matrix, 128 × 128; voxel size, 2.875 mm × 2.875 mm × 4 mm; axial slices, 35; FOV, 220 mm × 140 mm × 220 mm; TE, 35 ms; and TR, 3,000 ms.

### Image Preprocessing and Analyses

#### Vertex-Wise Analysis of Cortical Thickness

T1-weighted images (T1WI) were preprocessed using Freesurfer version 6.0.^1^ We reconstructed the cortical thickness maps to the fsaverage standard surface provided by Freesurfer; then, we determined the cortical thickness values in 68 bilateral Desikan–Killinay regions of interest (ROIs) in each participant (Desikan et al., 2006).

The preprocessed cortical thickness data were subjected to vertex-wise analysis using the mri-glmfit tool from Freesurfer. Cortical thickness was investigated using a generalized linear model, with age and sex as the covariates. To avoid false positives, a Monte Carlo simulation with 10,000 permutations, as implemented in Freesurfer [family-wise error (FWE), *p* < 0.01], was tested. Only those regions that survived these multiple comparisons are shown in the figures.

#### Tract-Based Spatial Statistics

Diffusion tensor imaging images were preprocessed using the FMRIB Software Library (FSL version 5.0.9^2^). In the preprocessing step, eddy current distortion and head motion for each raw DTI image were corrected using the eddy current function of the FSL. Individual brain binary masks were created using the Brain Extraction Tool with a fractional intensity threshold of 0.2, and an FA map was generated using DTIfit.

Tract-based spatial statistics (TBSS) were performed to calculate the ROI-specific mean FA value and explore whether there was any regional difference in the FA values between the groups of comparison (Smith et al., 2006). First, the FA maps of all participants were non-linearly aligned to the space of a study-specific template and registered to the Montreal Neurological Institute (MNI) coordinate space. Second, mean FA maps were created for every participant, and the mean FA skeleton was generated with a threshold FA > 0.02. Third, the aligned FA maps of each participant were projected onto the FA skeleton.

Using FSL randomization, permutation-based non-parametric *t*-statistics (10,000 permutations) were performed for group comparisons. A threshold-free cluster enhancement was used to correct multiple comparisons, and a significant difference between the groups at the cluster level was obtained at *p* < 0.001 (FWE-corrected) (Smith and Nichols, 2009). The WM was labeled with the reference atlas of ICBM-DTI-81 WM labels and the JHU WM tractography atlas supported by the FSL.

#### Network Construction and Network-Based Statistic Analysis

The rs-fMRI images were preprocessed using the CONN functional connectivity toolbox, version 20.b,^3^ from the SPM12 package.^4^ All preprocessing steps were performed using the CONN’s default preprocessing pipeline. In this preprocessing pipeline, the raw rs-fMRI images were realigned for motion correction, unwrapped, centered to (0, 0, 0) coordinates, corrected for slice-timing, and coregistered to each participant’s 3D T1WI. These images were then normalized to the MNI coordinate space, spatially smoothed with an 8-mm Gaussian kernel with full width at half maximum, and resliced into 2 × 2 × 2-mm voxels. Moreover, a default denoising pipeline from the CONN toolbox was also used. In this denoising pipeline, the preprocessed rs-fMRI images linearly regressed out the potential confounding effects of the blood-oxygen-level-dependent signal and temporal band-pass filtering with a band-pass filter of 0.008–0.09 Hz.

To define the network nodes, we used the FSL Harvard–Oxford atlas included in the CONN toolbox (Frazier et al., 2005; Desikan et al., 2006; Makris et al., 2006; Goldstein et al., 2007). For each participant, the average rs-fMRI time series of each ROI was extracted from 106 cortical and subcortical ROIs, and the Pearson’s correlation coefficients between the rs-fMRI time series of each ROI were calculated to construct the functional connectivity.

We used NBS to identify the functional networks associated with the BR; this is a nonparametric method based on the concept of cluster-based thresholding of statistical maps (Zalesky et al., 2010). First, a general linear regression was performed with the ROI-to-ROI matrix, and a statistical parametric map was created. Then, a threshold of *p* < 0.001 was applied to the statistical parametric map to extract highly associated connections and to identify the largest number of connected BR-associated networks. Finally, a permutation test (10,000 permutations) was performed to determine an empirical null distribution of the maximal BR networks, and an FDR-corrected *p*-value was assigned to each network. Networks with a corrected *p* < 0.05 were considered statistically significant. NBS analysis was performed using CONN (20.b).

### Calculation of the Behavioral Reserve Marker and the Relationship Between the Behavioral Reserve Marker and Reserve Proxies

Based on the original definition of reserve, we defined a BR marker as a residual of the difference between the actual behavioral score and the estimated score.


$$B\,e\,h\,a\,v\,i\,o\,u\,r\,a\,l\,R\,e\,s\,e\,r\,v\,e\,\left(B\,R\right)\,m\,a\,r\,k\,e\,r=B\,e\,h\,a\,v\,i\,o\,u\,r\,a\,l\,S\,c\,o\,r\,e_{o\,b\,s\,e\,r\,v\,e\,d}-B\,e\,h\,a\,v\,i\,o\,u\,r\,a\,l\,S\,c\,o\,r\,e_{e\,s\,t\,i\,m\,a\,t\,e\,d}$$

High BR marker values indicated a high BR, while low BR marker values indicated a low BR. Based on the median value of the BR marker, participants were divided into the high and low BR groups.

The estimated behavioral score was calculated based on the neuroimaging abnormalities and demographics. A general linear model was established using age, sex, cortical thickness, and the mean FA values to estimate the behavioral score. To reflect the specific neuropathological burden of FTD, we used the mean cortical thickness of the fronto-temporo-insular area (mean CTh_*FT*_) and the mean FA value of the frontotemporal WM (mean FA_*FT*_) (Du et al., 2007; Zhang et al., 2009; Pan et al., 2012).


$$B\,e\,h\,a\,v\,i\,o\,u\,r\,a\,l\,S\,c\,o\,r\,e_{e\,s\,t\,i\,m\,a\,t\,e\,d}=\alpha_{1}\times A\,g\,e+\alpha_{2}\times S\,e\,x+\alpha_{3}\times m\,e\,a\,n\,C\,T\,h_{F\,T}+\alpha_{4}\times m\,e\,a\,n\,F\,A_{F\,T}+\beta$$

After calculating the BR marker, we calculated the Pearson’s correlation coefficients between the BR marker and the previously noted reserve markers, such as the education level and occupational complexity used in CR in AD (Stern, 2012; Lee et al., 2019) and MR in PD (Chung et al., 2020a,b).

### Statistical Analysis

The Pearson’s correlation coefficients were calculated to assess the relationships among the continuous variables. A two-sample *t*-test was performed to compare variables between the high and low BR groups; two-tailed *p*-values < 0.05 were considered statistically significant.

To compare the slope differences between the two groups, we used the following formula (Kleinbaum et al., 2013):


Z⁢s⁢c⁢o⁢r⁢e=β1-β2S⁢t⁢a⁢n⁢d⁢a⁢r⁢d⁢e⁢r⁢r⁢o⁢r12-S⁢t⁢a⁢n⁢d⁢a⁢r⁢d⁢e⁢r⁢r⁢o⁢r22

Using this formula, we calculated the *Z* score using a *p*-value table. We used R studio (R studio, Boston, MA, United States) for statistical and graph visualization and BrainNet Viewer for network visualization (Xia et al., 2013).

## Results

### Demographics

The demographics and behavioral assessments of the participants and the two groups (low and high nBR) are summarized in Table 1. There were no differences in age, sex, education level, or occupational complexity between the high and low nBR groups. However, the FBI negative symptom scores were higher in the low nBR group than in the high nBR group (Table 1).

**TABLE 1:** Demographic characteristics and behavioral assessment of the participants with bvFTD classified into the low behavioral reserve for negative symptoms (nBR) group and the high nBR group.

	bvFTD (*n* = 40)	Low nBR (*n* = 20)	High nBR (*n* = 20)	*p*-Value
Demographics				
Diagnostic age, years	65.7 ± 8.4	65.1 ± 8.6	66.3 ± 8.4	0.656
Gender, women (%)	15 (37.5)	7 (35.0)	8 (40.0)	0.756
Level of education, years	11.9 ± 5.4	12.0 ± 5.9	11.7 ± 5.1	0.852
Occupational complexity (DOT rating)*^a^*	13.3 ± 4.6	13.3 ± 5.1	13.4 ± 4.2	0.927
Behavioral assessment				
FBI_*total*_	27.8 ± 11.9	35.0 ± 10.9	20.7 ± 7.9	<0.001
FBI_*negative*_	19.1 ± 7.9	24.4 ± 5.6	13.8 ± 6.2	<0.001
FBI_*positive*_	8.8 ± 6.9	10.7 ± 8.2	7.0 ± 4.8	0.094
MRI-FBI interval, days	28.9 ± 136.8	45.4 ± 120.3	12.5 ± 153.0	0.454

*^a^Occupational information of one participant classified into the high nBR group was not available.*

*bvFTD, behavioral variant frontotemporal dementia; DOT, Dictionary of Occupational Titles; FBI, frontal behavioral inventory.*

### Behavioral Scores and Neuroimaging Correlates

**FIGURE 2:**
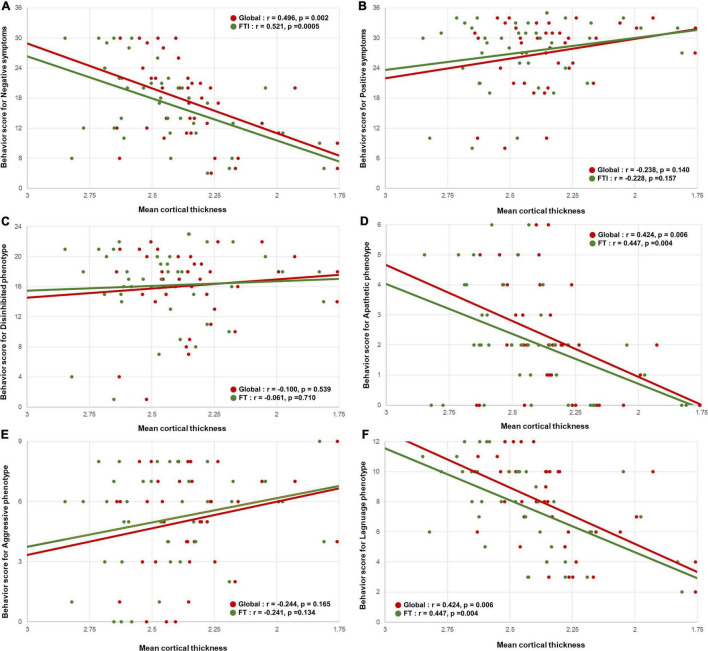
Correlation between the mean cortical thickness and the behavioral scores. Scatterplots showing the relationship between the behavioral scores for negative symptoms and the behavioral variant frontotemporal dementia neuropathology imaging biomarkers. **(A)** The behavioral scores for negative symptoms were positively correlated with the global mean cortical thickness and fronto-temporo-insular mean cortical thickness. **(B)** The behavioral scores for positive symptoms were not correlated with the global mean cortical thickness and fronto-temporo-insular mean cortical thickness. In phenotype aspects. **(D,F)** The behavioral scores for apathetic and language phenotypes were positively correlated with the global mean cortical thickness and fronto-temporo-insular mean cortical thickness. **(C,E)** The behavioral scores for disinhibited and aggressive phenotype were not correlated with the global mean cortical thickness and fronto-temporo-insular mean cortical thickness.

The behavioral scores for negative symptoms were positively correlated with the global (*r* = 0.496, *p* = 0.002) and fronto-temporo-insular (*r* = 0.521, *p* = 0.0005) mean cortical thicknesses (Figure 2A). However, the behavioral scores for the positive symptoms were not correlated with the global (*r* = −0.238, *p* = 0.140) and fronto-temporo-insular (*r* = −0.228, *p* = 0.157) mean cortical thicknesses (Figure 2B).

**FIGURE 3:**
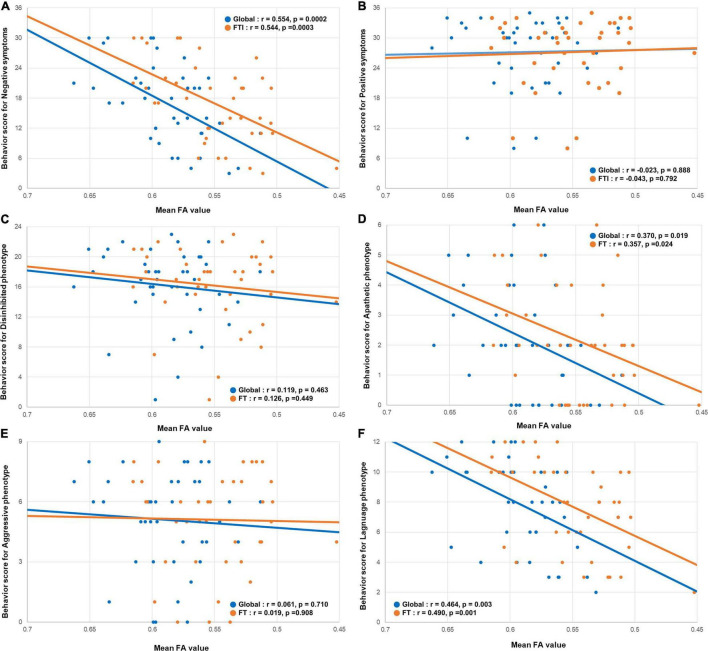
Correlation between the mean FA value and the behavioral scores. Scatterplots showing the relationship between the behavioral scores for negative symptoms and the behavioral variant frontotemporal dementia neuropathology imaging biomarkers. **(A)** The behavioral scores for negative symptoms were positively correlated with the global mean FA, and mean FA value of the frontotemporal WM. **(B)** The behavioral scores for positive symptoms were not correlated with the global mean FA, and mean FA value of the frontotemporal WM. In phenotype aspects. **(D,F)** The behavioral scores for apathetic and language phenotypes were positively correlated with the global mean FA, and mean FA value of the frontotemporal WM. **(C,E)** The behavioral scores for disinhibited and aggressive phenotype were not correlated with the global mean FA, and mean FA value of the frontotemporal WM. FA, fraction anisotropy; WM, white matter.

The behavioral scores for the negative symptoms were positively correlated with the mean FA values of the global (*r* = 0.554, *p* = 0.0002) and frontotemporal (*r* = 0.544, *p* = 0.0003) WM (Figure 3A). Similar to the cortical thickness, the behavioral scores for the positive symptoms were not correlated with the mean FA values of the global (*r* = −0.023, *p* = 0.888) or frontotemporal (*r* = −0.0043, *p* = 0.792) WM (Figure 3B).

Among the four phenotype subgroups, the apathetic phenotype was positively correlated with the global (*r* = 0.424, 0 = 0.006) and fronto-temporo-insular (*r* = 0.447, *p* = 0.004) mean cortical thicknesses. Furthermore, the language phenotype was also positively correlated with the global (*r* = 0.527, *p* = 0.0005) and fronto-temporo-insular (*r* = 0.574, *p* = 0.0001) mean cortical thicknesses (Figures 2D,F). However, the global (*r* = −0.100, *p* = 0.539) and fronto-temporo-insular (*r* = −0.061, *p* = 0.710) mean cortical thicknesses were not associated with the disinhibited and aggressive phenotypes (global: *r* = −0.224, *p* = 0.165; fronto-temporo-insular: *r* = −0.241, *p* = 0.134; Figures 2C,E). FA maps revealed that the apathetic phenotype was positively correlated with the mean FA values of the global (*r* = 0.370, 0 = 0.019) and fronto-temporo-insular (*r* = 0.357, *p* = 0.024) WM. Furthermore, FA maps also revealed that the language phenotype was positively correlated with the mean FA values of the global (*r* = 0.464, *p* = 0.003) and fronto-temporo-insular (*r* = 0.490, *p* = 0.001) mean cortical thicknesses (Figures 3D,F). However, the mean FA values of the global (*r* = 0.119, *p* = 0.463) and fronto-temporo-insular (*r* = 0.126, *p* = 0.449) mean cortical thicknesses were not associated with the disinhibited and aggressive phenotypes (global: *r* = 0.061, *p* = 0.710; fronto-temporo-insular: *r* = 0.019, *p* = 0.908; Figures 3C,E).

### Calculation of the Behavioral Reserve Marker and Its Relationship With the Reserve Proxies

We only used the negative symptom score for the BR because the positive symptoms did not show any significant correlation. As seen in Table 2, the general linear model demonstrated that the estimated behavioral score for negative symptoms was predicted by the fronto-temporo-insular cortical thickness (β = 10.504, *p* = 0.049) and the FA value of the frontotemporal WM (β = 75.919, *p* = 0.035). The *R*^2^ value of the model was 0.386 (adjusted *R*^2^ = 0.316; *F*-test: *p* = 0.002).

**TABLE 2:** General linear model to predict the behavioral score for negative symptoms.

Variables	β	Standard error	*p*-Value
Intercept	–42.788	19.550	0.035
Age	–0.117	0.132	0.379
Sex	0.134	2.311	0.954
FTI cortical thickness	10.504	5.140	0.049
FT FA	75.919	34.618	0.035

*FTI, fronto-temporo-insular area; FT, frontotemporal area; FA, fraction anisotropy.*

The apathetic and language phenotypes, for which significant correlations were noted between the behavioral scores and the neuroimaging abnormalities, were used for building a general linear model. Conversely, the disinhibited and aggressive phenotypes, for which no such significant correlations were noted, were not used. The general linear model demonstrated that the estimated behavioral score for the language phenotype was predicted by the fronto-temporo-insular cortical thickness (β = 4.779, *p* = 0.013) and the FA value of the frontotemporal WM (β = 25.891, *p* = 0.043). The *R*^2^ value of the model was 0.470 (adjusted *R*^2^ = 0.437; *F*-test, *p* = 0.0004). However, the general linear model for the apathetic phenotype did not identify a significant model (adjusted *R*^2^ = 0.221; *F*-test: *p* = 0.061).

**FIGURE 4:**
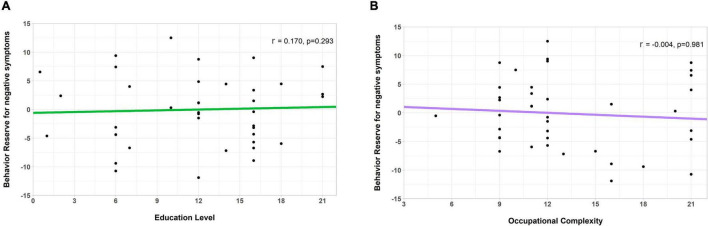
Correlation of the behavioral reserve marker for negative symptoms (an nBR marker) with the education level and occupation complexity (markers for other reserves). Scatterplots showing the relationship between the nBR marker, education level, and occupational complexity. The nBR marker was not significantly correlated with **(A)** the education level (*r* = 0.170, *p* = 0.293) or **(B)** the occupational complexity (*r* = –0.004, *p* = 0.981). nBR, behavioral reserve for negative symptoms.

After calculating the individual nBR markers, we calculated the relationships between the BR marker and the previously noted reserve proxies, such as the education level and occupational complexity used in CR in AD (Stern, 2012; Lee et al., 2019) and MR in PD (Chung et al., 2020a,b). As seen in Figure 4, the nBR marker was not correlated with the education level (*r* = 0.170, *p* = 0.293) or the occupational complexity (*r* = −0.004, *p* = 0.981). The three items of the DOT score did not correlate with the nBR marker (Supplementary Figure 1).

### Validation of the Behavioral Reserve for Negative Symptoms Marker

**FIGURE 5:**
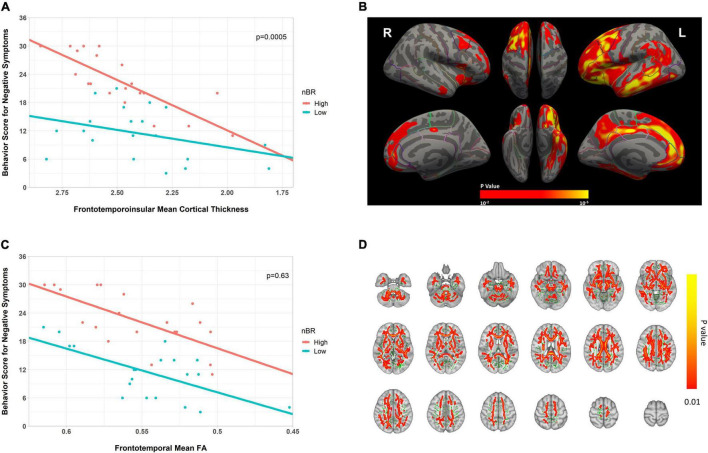
Scatter plot for the interaction of the behavioral reserve marker for negative symptoms (nBR) and the neuroimaging abnormalities (cortical thickness and FA) on behavioral score for negative symptoms in bvFTD. **(A,C)** Scatter plots for the interaction of the nBR with **(A)** fronto-temporo-insular mean cortical thickness and **(C)** frontotemporal WM FA on behavioral score for negative symptoms in bvFTD. For illustration, groups with high and low nBR markers (defined using the median value) are plotted separately. **(B)** Cortical thickness-related nBR, adjusted for the age and sex (*p* < 0.01; corrected at the cluster level at *p* < 0.01). **(D)** FA-related nBR (in red-yellow), adjusted for the age and sex, and the mean FA skeleton (in green, FA 0.2). The results are corrected by threshold-free cluster enhancement and 10,000 permutations. Cluster significance was tested at *p* < 0.01 and subjected to multiple corrections. nBR, behavioral reserve for negative symptoms; FA, fractional anisotropy; bvFTD, behavioral variant frontotemporal dementia.

We compared the relationship of the behavioral score for negative symptoms with the neuroimaging abnormalities between the high and low nBR groups. Compared to the low nBR group, the high nBR group showed a higher behavioral score (fewer symptoms) at a relatively normal cortical thickness and a steeper slope (*p* = 0.0005; Figure 5A). After adjusting for the age and sex, vertex-analysis of the cortical thickness revealed that the nBR marker was positively associated with the cortical thicknesses of the following: left superior frontal gyrus; middle frontal gyrus; orbitofrontal cortex; frontal opercularis; frontal triangularis; frontal orbitalis; middle temporal cortex; inferior temporal cortex; inferior parietal cortex; supramarginal gyrus; cingulate; precuneus; and right superior frontal, caudal middle frontal, medial orbitofrontal gyri (Figure 5B). Furthermore, a comparison of the FA values revealed that compared to the low nBR group, the high nBR group showed a higher behavioral score; however, the two groups had similar slopes (*p* = 0.63; Figure 5C). The TBSS of the FA value showed that the BR marker was associated with the FA value of the overall WM area, except in the sensory-motor cortices and occipital lobes (Figure 5D).

Regarding the language related BR (l-BR), the language scores were higher in the l-BR group than in the low l-BR group; however, the two groups had similar slopes for the fronto-temporo-insular mean cortical thickness and the frontotemporal WM FA value. Inter-group comparisons of the cortical thickness or FA maps did not identify any significant region.

### Identification of the Behavioral Reserve for Negative Symptoms Network

**FIGURE 6:**
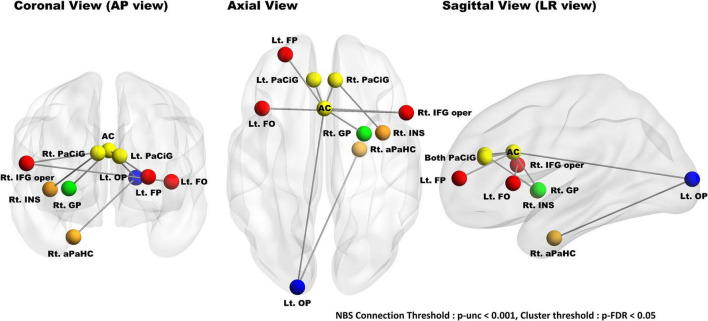
Results of group comparisons (high nBR > low nBR) with a network-based statistical analysis. NBS analysis identified a single nBR network composed of the cingulate gyrus (anterior division), paracingulate gyri, left frontal pole, left frontal operculum cortex, left occipital pole, right pallidum, right insular cortex, left anterior parahippocampus, and right inferior frontal gyrus (pars opercularis); the connection threshold was *p* < 0.001 (uncorrected), while the cluster threshold was *p* < 0.05. The nodes located in the frontal, temporal, cingulate, basal ganglia, and occipital lobes are marked red, orange, yellow, green, and blue, respectively. NBS, network-based statistical; nBR, behavioral reserve for negative symptoms; Lt., left; Rt., right; FP, frontal pole; FO, frontal operculum cortex; PaCiG, paracingulate gyrus; AC, cingulate gyrus, anterior division; GP, globus pallidus; IFG oper, inferior frontal gyrus pars opercularis; INS, insular cortex; aPaHC, parahippocampal gyrus; OP, occipital pole.

During group comparison, NBS analysis identified a single brain network (high nBR > low nBR) consisting of the anterior cingulate gyrus, paracingulate gyri, left frontal pole, left frontal operculum cortex, left occipital pole, right pallidum, right insular cortex, left anterior parahippocampal gyrus, and right inferior frontal gyrus (pars opercularis). The uncorrected connection threshold was *p* < 0.001, and the FDR-corrected cluster threshold was *p* < 0.05 (Figure 6). There was no functional connectivity related l-BR *via* NBS analysis.

## Discussion

In this study, we have proposed a concept of BR and investigated the neural correlates of BR in bvFTD, based on the definition of reserve (i.e., the discrepancy between the pathological burden and clinical manifestation) (Stern, 2012; Barulli and Stern, 2013). We hypothesized that the presence of BR explains the differences in the behavioral performances, despite similar pathological burdens, among participants with bvFTD. First, the nBR was defined, calculated, and validated. We then identified an interaction between the nBR marker and the neuroimaging abnormalities and its effect on behavioral performance. Finally, we identified the functional brain network associated with the nBR marker.

We wish to explain the individual variability of behavioral problems in bvFTD through the BR hypothesis. BR was considered to represent the difference between the estimated and observed behavioral performances. Our linear model calculated the estimated behavioral score from neuroimaging abnormalities. We used cortical thicknesses and FA values from DTI images to reflect the neuropathological burden of the GM and WM, respectively. Considering that AD-specific ROIs were more highly correlated with education (which is a proxy of CR) than with the global area (van Loenhoud et al., 2017), we additionally applied the neuropathological burden in the frontal, temporal, and insular lobes (these are representative pathological areas in bvFTD) (Du et al., 2007; Pan et al., 2012). Our experiments showed a higher correlation with these areas than with the global area. In the WM, the sagittal stratum (including the inferior longitudinal fasciculus and the inferior fronto-occipital fasciculus), cingulum (cingulate gyrus and hippocampus), fornix, stria terminalis, superior longitudinal fasciculus, superior fronto-occipital fasciculus, and uncinate fasciculus were selected as tracts of interest (Zhang et al., 2009).

The FBI scores and neuroimaging abnormalities were analyzed to create the model. The FBI score for negative symptoms was significantly correlated with the cortical thicknesses and FA values of the global and fronto-temporo-insular areas; however, the FBI score for positive symptoms was not. The cortical thickness and FA map-related FBI score for positive symptoms were considered to have no statistically significant areas because the difference in the scores between the participants was small. This can be attributed to the narrow distribution of the FBI scores for positive symptoms. Although the positive symptoms were more diagnostic for bvFTD, the negative symptoms were the most prominent symptoms at all stages (Benussi et al., 2021). According to the FBI manual, scores 25–30, 30–40, and >40 indicate mild, moderate, and severe disease states, respectively. The mean FBI score of our participants was 27.8; therefore, our data tended to represent participants with a mild disease status. In addition, the prevalence of genetic factors in bvFTD and AD is 30 and 5%, respectively (Rohrer et al., 2009). Because of the high proportion of genetic components, the environmental factors that are presumably core factors for BR may be relatively small.

The apathetic and language phenotypes (groups comprising negative items) also showed no significant correlations with neuroimaging abnormality. The apathetic phenotype included only two FBI items and excluded the major negative symptoms, such as indifference, disorganization, inattention, and personal neglect. The results for the language phenotype are thought to be because of the few language symptoms among the patients with FTD in this study, as only patients considered to have behavioral variants were selected and those considered to have semantic variants or progressive nonfluent aphasia were excluded. No significant correlations were observed between the nBR marker and the education level and occupation complexity, which were previously reported as proxies of other reserves in AD, FTD, and PD. In previous studies, only in cases with the disinhibited phenotype of bvFTD (which mostly consisted of the FBI score for positive symptoms), correlations between the education level and hypoperfusion in the frontotemporal area were observed on SPECT. However, apathic and language phenotypes, which were a part of the negative symptoms in the FBI, showed no correlated areas (Borroni et al., 2012; Maiovis et al., 2018). Our results were consistent with those of previous studies, suggesting that the education level may not be associated with BR.

Our major findings supported the BR hypothesis and demonstrated nBR marker-associated neuroimaging correlates. The high nBR group showed a higher behavioral score with lower neuroimaging abnormalities and a rapid decline of the behavioral score as reflective of cortical atrophy; this progressed similarly to CR in AD and MR in PD. Although our study is not a longitudinal study, the effect of the interaction between the nBR marker and neuroimaging abnormalities on the behavioral score was compatible with the traditional reserve concept (Stern, 2012; Barulli and Stern, 2013; Lee et al., 2019; Chung et al., 2020b). It has been reported that apathy is associated with the right dorsolateral prefrontal cortex, anterior cingulate, and putamen in FTD (Zamboni et al., 2008); attention is associated with the ventral prefrontal cortex in the attention-deficit/hyperactivity disorder (Durston et al., 2006); insights are associated with the orbitofrontal cortex and the frontal pole in the AD and FTD (Hornberger et al., 2014); and semantic performance is associated with the anterior temporal cortex in FTD (Williams et al., 2005).

The nBR marker was associated with the left frontal lobe, left precuneus, left cingulate, left middle and inferior temporal, left banks superior temporal, left supramarginal, and left inferior parietal cortical thicknesses. These areas overlapped with the previously reported areas associated with negative symptoms and further correlated with the default mode network (DMN). The DMN is divided into the ventral and dorsal medial prefrontal cortices (mPFCs), posterior cingulate, and precuneus (Raichle, 2015). The ventral mPFC is related to a personality change, emotional response, and mood control (Damasio, 1996; Bechara et al., 1997; Simpson et al., 2001). The dorsal mPFC is related to the regulation of emotional behavior and judgment of another person’s emotional state (Ochsner et al., 2004a,b).

Given that the negative symptoms of the FBI score are apathy, emotional flatness, inflexibility, personal neglect, and loss of insight, the results of these previous studies are in line with our results. The behavioral scores in those with lesser neuroimaging abnormalities were similar between the FA maps and cortical thickness analysis. Furthermore, as the FA value decreased, the corresponding decline in the behavioral score did not differ between the high and low BR groups. In FTD, structural connectivity is known to degrade prior to cortical atrophy (Gordon et al., 2016). Thus, nBR is more closely related to the GM. In addition, the BR, similar to other reserves, can be understood by two mechanisms: neural reserve and neural compensation (Stern, 2012). Neural reserve is a pre-existing brain network with a greater capacity to cope with a neuropathological burden; on the other hand, neural compensation is a brain network newly developed to compensate for the disruption of the pre-existing brain network due to the disease pathology. However, it remains unclear whether increased brain networks related to nBR in bvFTD are mainly associated with the capacity of neural reserve or neural compensation. From this hypothesis’s perspective, it can be interpreted that the GM of the high nBR group tolerated neurodegeneration better than the GM of the low nBR group (neural reserve mechanism). It can also be interpreted that compared to in the low nBR group, the WM in the high nBR group is better adapted to neurodegeneration by developing the compensation neural network identified in NBS analysis (neural compensation mechanism).

In addition, we also identified an nBR-related brain network (high nBR > low nBR) composed of the anterior cingulate gyrus, paracingulate gyri, left frontal operculum cortex, left occipital pole, right pallidum, right insular cortex, left parahippocampus, and right inferior frontal gyrus (pars opercularis). The anterior cingulate gyrus and the anterior insular cortices are a part of the salience network, and the paracingulate gyri, frontal operculum cortex, and pars opercularis of the inferior frontal gyrus are adjacent to the anterior cingulate and anterior insula. In bvFTD, salience network disruption is correlated with the clinical severity (Zhou et al., 2010; Day et al., 2013). Furthermore, the paracingulate gyri and the frontal pole were correlated with the recall performance in bvFTD. The functional connectivity between the occipital pole and the parahippocampus may be related to language ability in the FBI’s negative items.

Our study has several limitations. First, bvFTD has several genetic causes. Genetic perspectives include *C9orf72*, *MAPR*, and *GRN* genes, while proteinopathy includes the tau and TDP-43 proteins. However, we used a clinical diagnosis instead of a pathological or genetic-based diagnosis. Second, the neuropathological burden was calculated indirectly from MRI and did not include cellular or molecular data. Nevertheless, we used GM and WM to reflect the neuropathological burden as accurately as possible. Third, the estimated nBR using the residual approach could vary depending on which variables were used (Pettigrew and Soldan, 2019). Although we considered all available variables (age, sex, cortical thickness, and the mean FA values) and outcome measures (FBI score) in the model, we need to validate the nBR using another biomarker or outcome measure in the future. Finally, we could only suggest the effect of the interaction between nBR markers and neuroimaging abnormalities on the behavioral score of negative symptoms in this cross-sectional study. Hence, a longitudinal study would help confirm whether greater nBR would delay the decline of behavioral score or disease progression.

In conclusion, we propose a novel concept of BR in bvFTD, which is associated with the individual’s capacity against its neuropathological burden, especially for negative symptoms. The nBR marker-related GM areas and the functional brain networks were also found centered at the frontotemporal areas. Participants with a greater nBR marker would show lesser clinical manifestations of the same neuropathological burden. These findings can be used to predict the clinical progression of each individual with bvFTD, thus enabling physicians to provide appropriate interventions when available.

## Data Availability Statement

The raw data supporting the conclusions of this article will be made available by the authors, without undue reservation.

## Ethics Statement

The studies involving human participants were reviewed and approved by the Institutional Review Board of the Samsung Medical Center. The patients/participants provided their written informed consent to participate in this study.

## Author Contributions

SHK performed the statistical analyses. SHK, JHP, and YJ performed the study design. BHL and SWS performed the data collection. SHK, BHL, SWS, and YJ performed the drafting of the manuscript. SHK, YJK, and PL performed the imaging data processing and analysis. All authors contributed to the article and approved the submitted version.

## Conflict of Interest

The authors declare that the research was conducted in the absence of any commercial or financial relationships that could be construed as a potential conflict of interest.

## Publisher’s Note

All claims expressed in this article are solely those of the authors and do not necessarily represent those of their affiliated organizations, or those of the publisher, the editors and the reviewers. Any product that may be evaluated in this article, or claim that may be made by its manufacturer, is not guaranteed or endorsed by the publisher.

## Footnotes

1
https://surfer.nmr.mgh.harvard.edu

2
https://www.fmrib.ox.ac.uk/fsl

3
https://www.nitrc.org/projects/conn

4
https://www.fil.ion.ucl.ac.uk/spm

## Supplementary Material

Supplementary Figure 1 Relationship between the behavior reserve marker for negative symptoms (nBR marker) and occupation complexity. Scatterplots showing relationship between nBR marker and occupational complexity. nBR markers were not significantly correlated with **(A)** complexity of data (*R* = −0.013, *p* = 0.982), **(B)** complexity of people (*R* = 0.115, *p* = 0.779), and **(C)** complexity of things (*R* = −0.796, *p* = 0.118).

## Abbreviations

AD: Alzheimer’s Disease
BR: behavioral reserve
bvFTD: behavioral variant frontotemporal dementia
CR: cognitive reserve
DTI: diffusion tensor imaging
DOT: Dictionary of Occupational Titles
FEW: family-wise error
FA: fractional anisotropy
FBI: frontal behavioral inventory
FTD: frontotemporal dementia
GM: gray matter
MRI: magnetic resonance imaging
mean CTh_*FT*_: mean cortical thickness of the fronto-temporo-insular area
mean FA_*FT*_: mean FA value of the frontotemporal WM
MR: motor reserve
MNI: Montreal Neurological Institute
nBR: behavioral reserve for negative symptoms
NBSs: network-based statistics
PD: Parkinson’s Disease
ROIs: regions of interest
rs-fMRI: resting-state functional magnetic resonance imaging
SPECT: single-photon emission computed tomography
T1WI: T1-weighted images
TBSS: tract-based spatial statistics
WM: white matter.
