# Supplementary figures and images for: Behavioral Reserve in Behavioral Variant Frontotemporal Dementia
Source: Front Aging Neurosci. 2022 Jun 20;14:875589. doi: 10.3389/fnagi.2022.875589 (PMC9252599; doi:10.3389/fnagi.2022.875589)

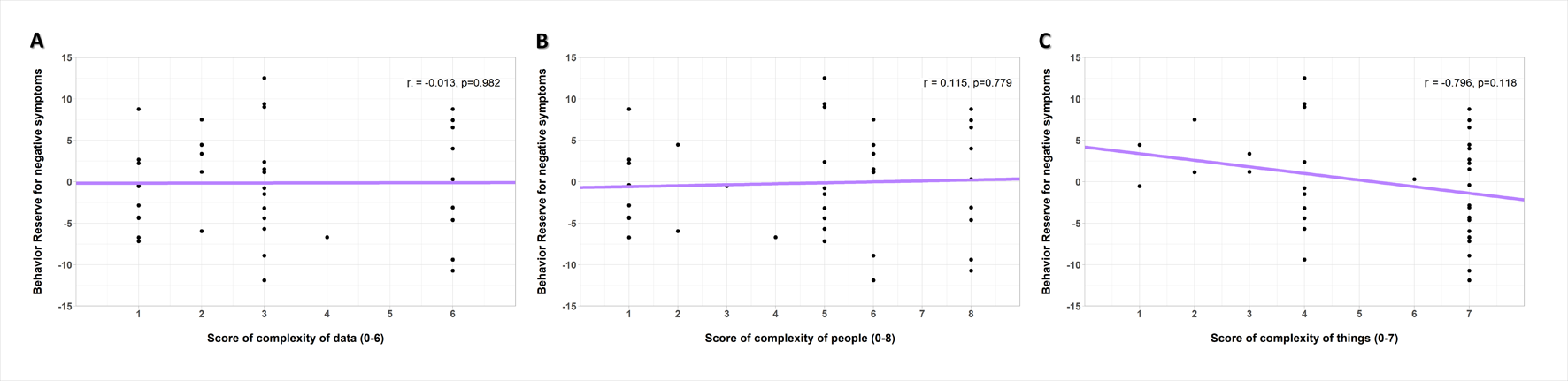

Supplement: Supplementary Figure 1 — Relationship between the behavior reserve marker for negative symptoms (nBR marker) and occupation complexity. Scatterplots showing relationship between nBR marker and occupational complexity. nBR markers were not significantly correlated with (A) complexity of data (R = −0.013, p = 0.982), (B) complexity of people (R = 0.115, p = 0.779), and (C) complexity of things (R = −0.796, p = 0.118). [file Image_1.TIF]
